# Engineering autonomous closed-loop designer cells for disease therapy

**DOI:** 10.1016/j.isci.2022.103834

**Published:** 2022-01-29

**Authors:** Mohamed Mahameed, Martin Fussenegger

**Affiliations:** 1 ETH Zurich, Department of Biosystems Science and Engineering, Mattenstrasse 26, CH-4058 Basel, Switzerland; 2 University of Basel, Faculty of Life Science, 4001 Basel, Switzerland

**Keywords:** Biopharmaceuticals, Molecular genetics, Synthetic biology

## Abstract

Synthetic biology has made it possible to engineer mammalian cells for on-demand delivery of therapeutic agents, providing therapeutic solutions for chronic or intractable diseases. Currently, most of the engineered therapeutic cells are regulated by the administration of exogenous inducers, but the need for repeated administration of these xenobiotics is problematic from the viewpoints of patients' compliance and quality of life, as well as possible side effects. Recently, synthetic biologists started to address these issues by constructing autonomous, closed-loop therapeutic cells, often referred to as designer cells. These cells are equipped with sensing modules that detect and link marker(s) of the target disease to signaling cascades that stimulate the secretion of specified therapeutic agents in a timely and quantitative manner, without the need of exogenous inducers. Here, we review recent work on designer cell engineering and their *in vivo* therapeutic applications, focusing on the molecular mechanisms and signaling pathways employed.

## Graphical Abstract

**Figure:**
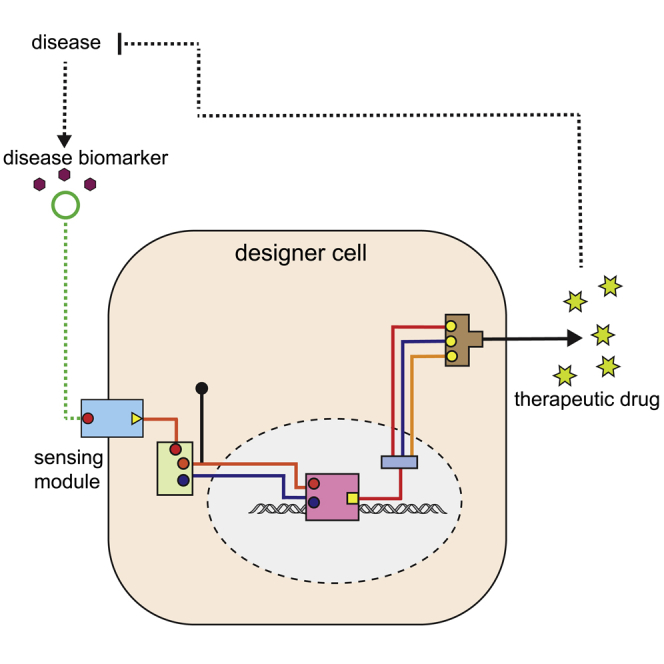

## Introduction

Cell-based therapy is opening up the possibility of novel therapeutic solutions for diseases such as organ failure and tissue dysfunction, where conventional drugs are of limited efficacy or impose a severe burden on the patients' quality of life. For example, cell-based alternatives to repetitive and invasive drug injections in patients with chronic diseases, such as diabetes, have been extensively investigated. Indeed, *in-vivo* implantation/injection of healthy or genetically modified cells has been considered to treat a range of diseases, including metabolic disorders, neurodegenerative diseases, genetic defects, and multiple types of malignancies (Buzhor et al., 2014; Filipovich, 2008; Kim and Keating, 2019; Yasuhara et al., 2017; Grupp et al., 2013). To date, the best-known clinical application is probably the use of chimeric antigen receptor (CAR)-T-cells to treat various types of blood cancers that are frequently refractory to standard chemotherapy (Grupp et al., 2013; Till et al., 2008).

**Figure 1:**
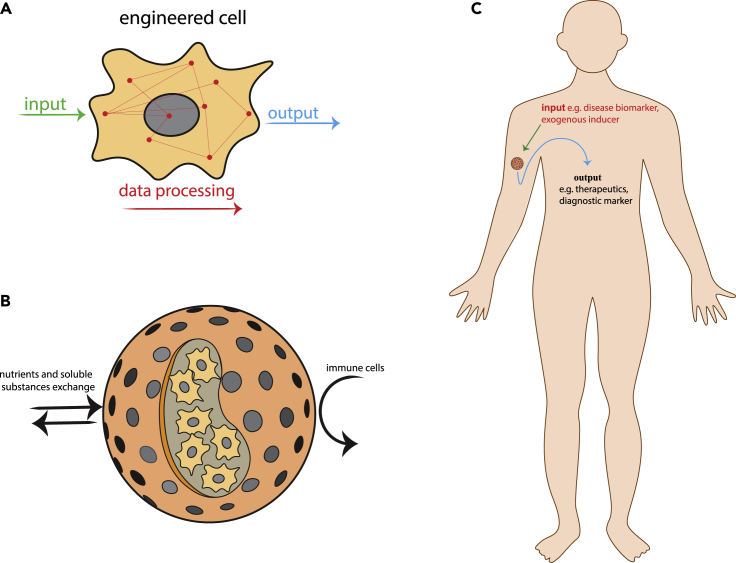
Schematic presentation of the engineering steps involved in the development of therapeutic cells (A) Mammalian cells are engineered with a variety of genetic elements, depending upon the desired cell performance. Upon receiving an activating signal input, these engineered cells initiate a cascade of biochemical reactions leading to a specific output. (B) Engineered cells are generally encapsulated in immune-protective shells, which shield them from immune system detection, while allowing transfer of nutrients and other substances. (C) The device is then implanted into the body to perform the designed therapeutic or diagnostic task.

Early work was focused on stem cell biology, based on the discovery of pluripotent stem cells (PSCs) by James Thomson and the later introduction of induced pluripotent stem cell (iPSC) technology by Shinya Yamanaka and John B. Gurdon (Thomson et al., 1998; Takahashi and Yamanaka, 2006). However, the clinical applicability of therapies based on stem cells has been severely limited by the risk of tumor formation because of undifferentiated or incompletely differentiated stem cells (Ota, 2008; Bailey, 2012; Kimbrel and Lanza, 2015; Einhorn and Iorio, 2017). For this reason, alternative strategies based on mammalian cells equipped with synthetic or modified genetic circuits and signaling networks have recently received more attention. In this approach, somatic cells are *ex vivo* engineered with a set of genes which enable biochemical linking of certain signal stimuli to a desired cellular output, such as the initiation of therapeutic protein secretion (Kojima et al., 2020; Scheller and Fussenegger, 2019; Heng et al., 2015) (Figure 1A). The engineered cells are then encapsulated with a semipermeable polymer to protect them from the host immune response, while allowing transfer of nutrients and other molecules (Figure 1B). Several types of immune-compatible polymers have recently been synthesized to provide *in vivo*-implanted cells with a protective and supportive shield suitable for long-term application (Rokstad et al., 2002; Ashimova et al., 2019). Following *ex vivo* encapsulation, the capsule device is introduced into the body, where the synthetically programmed cells mediate a specific therapeutic or diagnostic function (Figure 1C) (Sedlmayer et al., 2018; Teixeira and Fussenegger, 2017, 2019; Schukur and Fussenegger, 2016).

**Figure 2:**
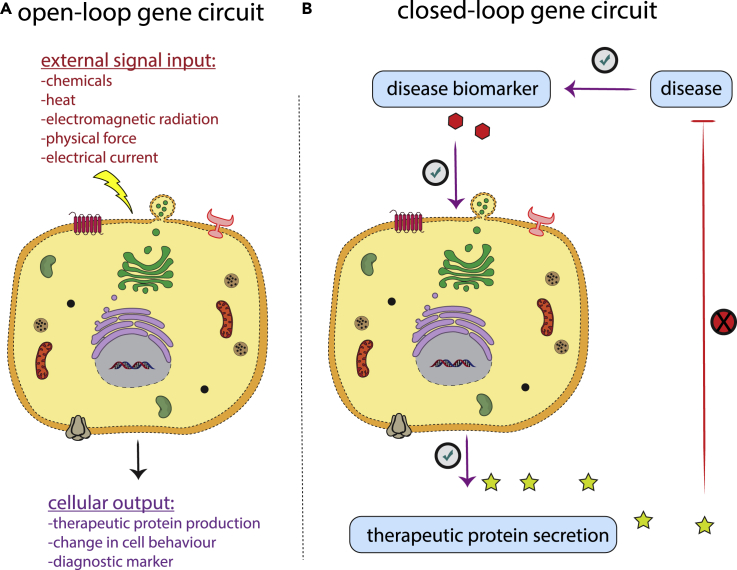
Mechanisms of action of therapeutic cells (A) Therapeutic cells with an open-loop gene circuit. An exogenous signal input is externally applied to activate the cellular machinery required for output production. (B) Therapeutic cells with a closed-loop gene circuit (designer cells). The close-loop gene circuit enables autonomous cell function. A defined disease biomarker is utilized as the signal initiator, and the output, which is generally a therapeutic protein, modifies the disease state. Alleviation of the disease state decreases the disease biomarker levels, thereby reducing the stimulation of the designer cells. This negative-feedback loop enables fine control of therapeutic output as a function of disease state/biomarker concentration in the circulation.

The earliest therapeutic cells were based on controllable, open-loop gene circuits, which require exogenous signaling initiators to switch the cells' behavior (Figure 2A). Various chemicals, heat, physical force, light, electricity, and even radio waves have been used as inducers (Figure 2B) (Doshi et al., 2020; Stefanov et al., 2021; Strittmatter et al., 2021; Müller et al., 2015; Krawczyk et al., 2020; Stanley et al., 2012). Although engineering open-loop systems is relatively straightforward, these systems have various drawbacks in a therapeutic setting. First, they rely on frequent delivery of the external inducer. Second, the functional behavior of open-loop systems is not readily predictable *in vivo* because of interindividual variability of physiological and pathological factors. Thus, tedious personal dosage adjustment for each patient may be required (Delcò et al., 2005; Verbeeck and Musuamba, 2009). In addition, light-controlled gene circuits can be problematic because of the limited penetration of light through living tissues (Ye and Fussenegger, 2019). However, the functional dependency of the open-loop expression systems on inducer availability in the cellular environment can be advantageous in certain circumstances. For example, open-loop operating devices can be easily shut down in the event of therapeutic-induced toxicity simply by stopping the administration of the inducer. In addition, personal dosage adjustment of the activating inducer may offer an additional control point between the typical ON/OFF endpoints, providing more flexibility to achieve an optimal individual response.

Recently, there has been increasing interest in the development of genetically engineered therapeutic cells that functionally mimic specialized cells in the body (Haellman and Fussenegger, 2016). These so-called designer cells are rationally engineered to continuously and accurately sense certain physiological inputs, integrate and process this biochemical information, and respond in a programmed manner (Figure 2B). These closed-loop feedback-controlled signaling systems enable the cells to respond specifically and quantitatively to changes in disease biomarker(s) in the blood, allowing precise and timely output control. Here, we review recent work on the engineering of designer cells, as well as their therapeutic applications in *in vivo* disease models.

## Designer cells for the treatment of metabolic and endocrine diseases

**Figure 3:**
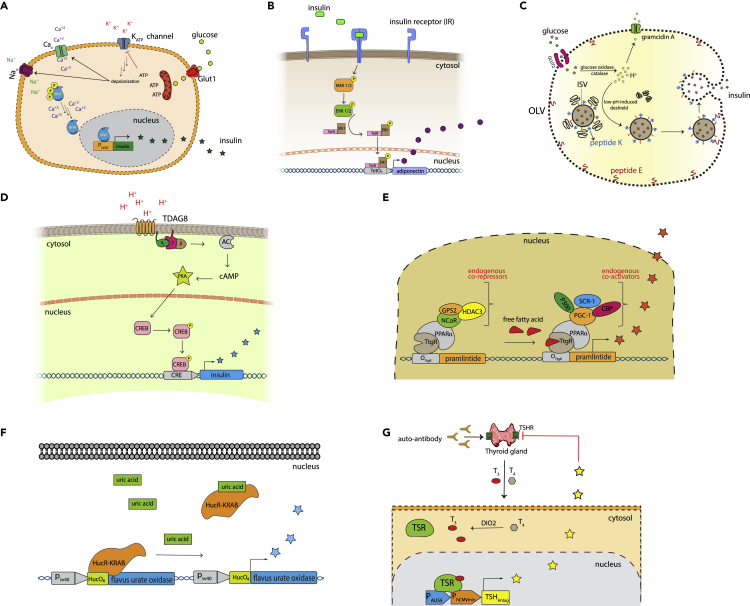
Designer cells to treat metabolic diseases (A) The engineering of pancreatic β-cell-mimicking designer cells. High levels of glucose cause overproduction of ATP, inducing closure of ATP-sensitive potassium channels. Accumulation of potassium ions in the cytoplasm causes mild membrane depolarization, which activates voltage-dependent calcium and sodium channels. These ion shifts lead to a high level of calcium in the cytosol, and this induces dephosphorylation of NFAT. Dephosphorylated NFAT is translocated to the nucleus to initiate gene expression. In this work, HEK-293T cells were ectopically engineered with a Cav1.3 channel and P_NFAT_-insulin expression cassette in order to link high glucose levels to insulin secretion. (B) The engineering of designer cells to overcome insulin resistance in peripheral tissues. HEK-293T cells were engineered with plasmids encoding to insulin receptor (IR), TetR-Elk1 fusion protein, and adiponectin under a TetO_7_ promoter. High insulin levels in the blood result in insulin binding to the IR receptor on the designer cell membrane and this activates the MAPK pathway, leading to phosphorylation of the TetR-Elk1 chimeric transcription factor. The phosphorylated transcription factor is translocated to the nuclease, and induces adiponectin gene expression. (C) The engineering of synthetic β-cell-like therapeutic machines for diabetes treatment. Liposome-based structures, called outer large vesicles (OLVs), have been chemically fabricated with GLUT2, gramicidin A, and peptide E anchored in the bilayer part. The luminal space of OLV contains soluble glucose oxidase, catalase, and insulin-loaded inner small liposomes (ISVs). Under normal conditions, the fusion of ISVs is prevented by steric hindrance involving the PEG–CDNA and GDNA–CH bulky groups. When the glucose concentration increases and glucose enters OLVs through GLUT2, it is enzymatically converted to gluconic acid by glucose oxidase. The resulting high proton levels decouple PEG–CDNA GDNA–CH aggregation, which permits the formation of coiled-coil interactions between peptide K and peptide (E) These strong protein-protein interactions eventually enforce IVS fusion and thereby release insulin. (D) The development of designer cells to manage ketoacidosis. HEK-293T cells were engineered with TDAG8, a pH-sensitive G protein-coupled receptor (GPCR). High levels of hydrogen ions stimulate TDAG8 and activate the adenylate cyclase pathway. The production of the second messenger cAMP activates protein kinase A (PKA), which phosphorylates cAMP response element-binding protein (CREB). Phosphorylated CREB binds to its response element, CRE, promoting insulin transcription. (E) Free fatty acids (FFA)-regulated designer cells for management of diet-induced obesity. The synthetic transcription factor, TtgR-PPARα, binds to the TtgR response sequence and represses gene expression. At high concentrations, FFAs bind to TtgR-PPARα and elicit conformational change, leading to the recruitment of cellular coactivators that facilitate pramlintide gene expression. (F) The engineering of urate homeostasis-restoring designer cells. HEK-293T cells were equipped with a chimeric repressor consisting of bacterial repressor HucR fused with a Krüppel associated box (KRAB) domain, which functions as transcriptional repressor in eukaryotes. The cells were also engineered to express flavus urate oxidase gene under the P_sv40_-HucO_7_ promoter. Under normal conditions, the HucR-KRAB chimera binds to the HucO_7_ sequence and strongly represses gene expression. High urate levels trigger dissociation of HucR-KRAB, leading to increased gene expression. (G) The engineering of designer cells for treatment of Graves' disease. In Graves' disease, autoantibodies are produced against thyroid-stimulating hormone receptors (TSHR), leading to overstimulation of the thyroid gland and excessive release of thyroid hormones, T_3_ and T_4_. In this study, mammalian cells were equipped with a synthetic thyroid-sensing receptor (TSR) consisting of Gal4 DNA-binding protein and human thyroid receptor α. When thyroid hormone levels increase, the TSR binds to the P_AUS_ sequence located upstream of P_hCMVmin_, resulting in expression of TSH-_Antag_, a competitive protein-based inhibitor of THSR in the thyroid gland.

Diabetes mellitus (DM) affects more than 45 million people worldwide (Kharroubi, 2015). Patients diagnosed with DM experience chronically high blood glucose levels, which may lead to life-threatening complications in the absence of medical intervention (Melendez-Ramirez et al., 2010; Papatheodorou et al., 2018). Type-1 DM is a consequence of autoimmune destruction of insulin-secreting pancreatic β-cells, whereas type-2 DM involves insulin resistance of the peripheral tissues (Banday et al., 2020). Therapeutically, insulin injection is the main treatment for type-1 DM and for complicated cases of type-2 DM (Silver et al., 2018). However, repeated injections are invasive and tend to reduce the patients' compliance and quality of life. Therefore, efforts have been made to develop synthetic β-cell-mimicking designer cells. Xie et al. linked high glucose levels to insulin secretion using non-endocrine designer cells, and showed that their strategy worked in a DM mouse model (Xie et al., 2016). They utilized ATP overproduction in response to high intracellular glucose levels as the input signal (DM biomarker) (Figure 3A). Elevated levels of ATP in the cytoplasm lead to closure of the ATP-sensitive potassium channel (K_ATP_), inducing a transient cellular depolarization that is further amplified by the opening of voltage-dependent calcium and sodium channels, resulting in a significant increase of intracellular calcium concentration. The resulting increase of stored cytoplasmic calcium was linked to P_NFAT_-driven insulin transcription through nuclear translocation of a calcium-responsive transcription factor. These cells proved effective to restore glucose homeostasis in a type-1 DM mouse model. Ye et al. engineered therapeutic designer cells that sense high insulin levels and respond by producing adiponectin, which is a protein hormone that reverses insulin resistance and restores glucose homeostasis and a normal lipid profile (Ye et al., 2017). In their work, HEK-293T cells were ectopically engineered with insulin receptor (IR) and a chimeric transcription factor consisting of tetracycline repressor, TetR, fused to the human ELK1-derived transactivation domain (TetR-Elk1) (Figure 3B). When insulin binds to IR, it initiates a series of signal transduction cascades that result in the phosphorylation of the TetR-Elk1 hybrid. The phosphorylated TetR-Elk1 is then shuttled to the nucleus, where it induces adiponectin expression through a P-_TetR_-driven promoter. These designer cells reverse insulin resistance and prevent lipid-caused complications in an ob/ob mouse model.

Totally synthetic “β-cells”, also called artificial beta cells (AbCs), have been developed as “sense-reactive” designer cells for diabetes management (Chen et al., 2018). In this work, Chen et al. chemically constructed liposome-based vesicles-in-vesicle superstructures, which are equipped with the molecular machinery needed for mimicking β-cells, by synthesizing insulin-containing inner small liposomes (ISVs), similar to insulin storage granules in mature β-cells (Figure 3C). These ISVs are encapsulated with a bigger bilayer, called the outer large vesicle (OLV), to form a synthetic plasma membrane. Glucose transporter 2 (GLUT2) is anchored to OLVs in order to allow glucose shuttling. In addition, they placed glucose oxidase and catalase in the ”cytoplasmic” part of OLVs to enable the conversion of glucose molecules to gluconic acid. Overall, these reactions lead to an increase of acidity inside the intra-OLV compartment as a function of glucose level. Gramicidin A was also inserted in the OLV membrane, in order to buffer the proton concentration over time. Mechanistically, high glucose levels inside OLVs causes a dramatic decrease of pH, which induces dehybridization of the polyethylene glycol-conjugated cytosine-rich DNA (PEG–CDNA) and the cholesterol-ended guanine-rich DNA (GDNA–CH) anchored on the ISVs (shown in black and orange). The proton-induced dehybridization serves to deshield peptide K, sterically exposing it to peptide E, an anchor protein constitutively localized in the inner surface of the OLV. Coiled-coil interactions between these peptides force the fusion of ISVs and thereby release insulin. This system has been successfully tested in an *in vivo* mouse model, with promising results. However, despite its ability to autonomously regulate glucose levels, this device has to be administered frequently as it lacks the ability to replenish empty ISVs with new insulin cargo.

Ketoacidosis (DKA) is a serious hyperglycemic emergency in people with DM, especially type-1 DM (Dhatariya et al., 2020). This life-threatening metabolic condition is because of a drastic reduction or total loss of circulating insulin, leading to the overproduction of acidic ketone bodies and a marked decrease of blood pH (Rosival, 2015). The most effective treatment of this rapidly deteriorating metabolic disturbance is insulin injection. Ausländer et al. developed designer HEK-293T cells, called HEK_pH-Guard_, which could effectively reverse KDA in a type-1 DM mouse model (Ausländer et al., 2014). HEK_pH-Guard_ cells are engineered to express TDAG8, which is a G protein-coupled receptor (GPCR) that senses extracellular hydrogen ions. Stimulation of TDAG8 elicits a signaling cascade that leads to the phosphorylation of cAMP-responsive element-binding protein 1 (CREB1). Activated CREB1 is translocated to the nucleolus, where it binds to a synthetic promoter (P_CRE_) containing cAMP-response elements (CRE) and serves as a transcription factor. Thus, the authors genetically linked the increase in hydrogen ion concentration (a biomarker of KDA) to secretion of insulin in HEK_pH-Guard_ cells (Figure 3D).

Obesity is a common metabolic disorder (Chooi et al., 2019), and is associated with the development of cardiovascular disease, diabetes, and cancer (Blüher, 2019). Although obesity can be prevented or reversed by lifestyle changes, such as restriction of dietary intake and physical exercise, many obese patients still require medical treatment. Currently, the main treatment options are anti-obesity medications, which are generally only partially effective, or bariatric surgery (Ruban et al., 2019), which can lead to serious and sometimes fatal complications. Rösgger et al. engineered designer cells that can ameliorate diet-induced obesity in mice (Rössger et al., 2013). They utilized HEK-293T cells in which stable expression of a synthetic free fatty acid-induced transcription factor is biochemically connected to the secretion of pramlintide, a clinically licensed appetite-suppressing peptide hormone (Figure 3E). The engineered transcription factor is a chimeric protein consisting of a phloretin-responsive repressor (TtgR) fused to peroxisome proliferator-activated receptor-α (PPARα). Exposure of the cells to high blood levels of free fatty acids releases the inhibitory effects of various endogenous repressors on the PPARα chimera, leading to expression of pramlintide via a TtgR-specific promoter.

Elevated uric acid levels, known as hyperuricemia, cause multiple metabolic disorders such as gout and tumor lysis syndrome (Skoczyńska et al., 2020). Uric acid, a by-product of purine metabolism, is barely soluble in aqueous environments, and high levels of this compound tend to deposit as crystals in joints, kidneys, and other tissues in the body. Kemmer et al. constructed a synthetic, cell-based system based on a modified *Deinococcus radiodurans*-derived protein that senses uric acid levels and elicits the secretion of engineered *Aspergillus flavus* urate oxidase, which promotes uric acid excretion (Kemmer et al., 2010). This closed-loop system incorporates a bacterial transcriptional repressor (HucR) with high affinity for a DNA sequence motif (hucO) in a uric acid-free environment (Figure 3F). In the presence of uric acid at a sufficient concentration, HucR dissociates from DNA, leading to initiation of gene expression. To further enhance the repression, HucR was C′-terminally fused to a Krueppel-associated box (KRAB) domain. This device successfully restored the urate concentration to normal levels in urate oxidase-deficient mice.

Graves' disease is an autoimmune disorder caused by the generation of autoantibodies that stimulate thyroid-stimulating hormone (TSH) receptors in the thyroid gland, leading to constitutive and chronic over-secretion of these hormones (T_3_ and T_4_) into the blood (Davies et al., 2020). Current therapeutic interventions include anti-thyroid medications, radioiodine treatment, and in some cases thyroidectomy followed by administration of exogenous thyroid hormones. Saxena et al. engineered designer cells equipped with a self-regulating sensor–effector TSR-TSH_Antag_ circuit (Saxena et al., 2016). This system contains a chimeric protein consisting of the ligand-binding domain of TR fused with Gal-4, a yeast DNA-binding domain (Figure 3G). In the presence of high circulating levels of thyroid hormones T_4_/T_3_, this hybrid protein binds to Ga-l4 response element and initiates a complex transcription process involving steroid receptor coactivator-1 (SRC-1) and TRAP 220, thereby promoting histone acetylation and resulting in gene expression. As a therapeutic output, they employed thyroid-stimulating hormone receptor antagonist (TSH_Antag_), which competitively inhibits TSHR in the thyroid gland, thus decreasing the production of thyroid hormone T_3_/T_4_ and alleviating the disease state.

## Designer cells with antimicrobial functionality

**Figure 4:**
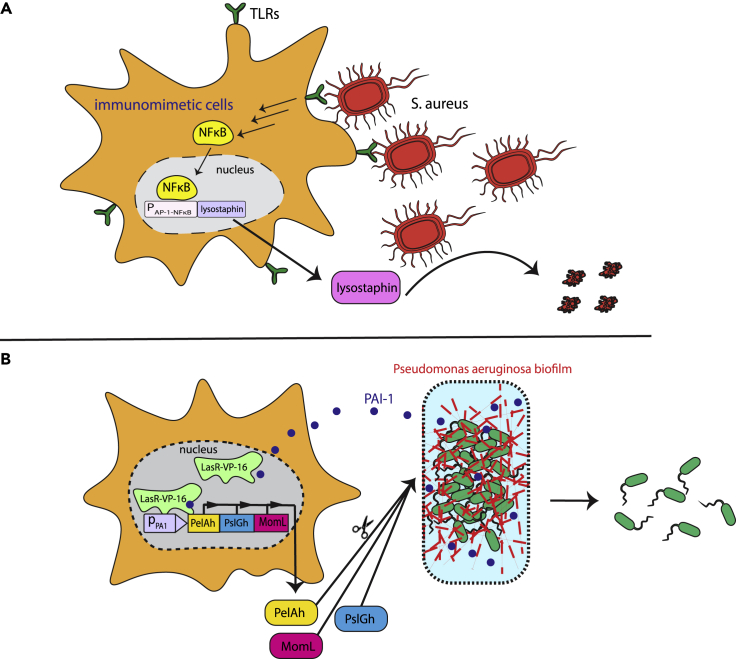
Designer cells to treat drug-resistant infections (A) The engineering of immunomimetic cells to deal with difficult-to-treat *S. aureus* infection. HEK-293T cells were equipped with toll-like receptors (TLRs) that recognize bacterial epitopes on the membranes. Binding of TLRs to bacterial cell-anchored molecules induces a signal cascade leading to activation of NF-κB, which is wired to expression of the bactericidal protein lysostaphin. (B) Designer cells for eradication of biofilm-forming *Pseudomonas aeruginosa*. Cells were equipped with a PAI-1-regulated LasR-VP16 transactivator. Bacteria inside the biofilm produce the signaling molecule PAI-1, which assists them to evade the immune system. The binding of PAI-1 activates LasR-VP16, leading to expression of the synergistically acting biofilm-dissolving proteins PelAh, PslGh, and MomL.

It has been estimated that more than 15 million people die each year from infectious diseases or their complications (Ahrens and Pigeot, 2014). Currently, the main treatment for bacterial infections is antibiotics, but drug resistance is becoming an ever-increasing problem (Aslam et al., 2018). Recently, synthetic biologists have started to develop engineered mammalian cells designed specifically to eliminate multidrug-resistant (MDR) organisms. Liu and coworkers focused on designer cells with the ability to efficiently detect and eradicate methicillin-resistant *Staphylococcus aureus* (MRSA) – a difficult-to-treat superbug (Liu et al., 2018). They engineered HEK-293T cells expressing toll-like-receptors (TLRs) that recognize various epitopes presented on the bacterial membrane. When these immunomimetic cells encounter and bind to bacteria, they are triggered to secrete lysostaphin, a bacteriolytic enzyme highly lethal to *S. aureus* (Figure 4A). The binding of TLRs to bacterial membrane-anchored molecules activates NF-κB signaling, resulting in the expression of a therapeutic gene, in this case lysostaphin, under an NF-κB-responsive promoter. These designer cells were highly effective in treating MRSA infections both *in vitro* and in a mouse model.

Another strategy is to focus on biofilm disintegration. Biofilm is a complex structure composed of aggregates of bacterial cells interconnected by endogenously produced polymers (Flemming et al., 2016). Bacterial strains that form biofilms are generally hard to treat, since they can physically evade the immune system because of the cross-linked jelly-like environment of the biofilm, which also restricts the access of antibiotics. The consequent continuous exposure to subtherapeutic levels of antibiotics further increases the resistance of the bacteria. Thus, targeting the biofilm matrix seems a promising approach to treat MDR strains. Sedlmayer et al. engineered nonimmune designer cells designed to selectively block *Pseudomonas aeruginosa* biofilm formation (Sedlmayer et al., 2017). They provided HEK-293T cells with a synthetic gene network harboring a PAI-1-activated transcription factor. PAI-1 is a bacterial autoinducer secreted by *Pseudomonas aeruginosa* that is involved in cellular communication (quorum sensing); it assists the bacteria to evade the immune response (Figure 4B). This sensor-transcription hybrid consists of *P. aeruginosa* LasR-derived activator fused to VP-16 transcriptional transactivator. The exposure of these quorum-quencher cells to *P. aeruginosa* PAI-1 triggers transcription of genes controlled by a LasR-response element. They utilized three biofilm-dissolving outputs, glycoside hydrolases PslGh and PelAh, and autoinducer-inactivating lactonase (MomL), with the aim of achieving synergistic action. The results suggest that this is a promising approach for the treatment of infections involving biofilm-forming bacteria.

## Designer cells to treat inflammatory diseases

**Figure 5:**
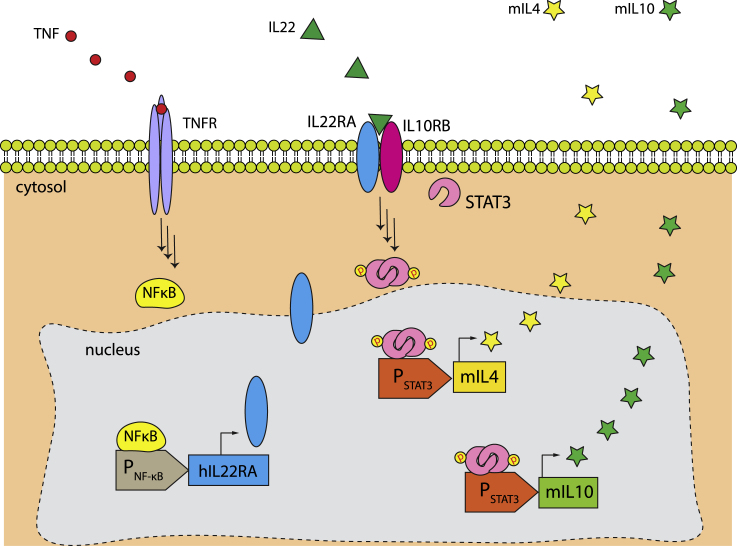
Designer cells for the management of psoriasis (A) The design of autonomous therapeutic cells with an AND-ON gate for psoriasis treatment. Cells were equipped with TNF receptor (TNFR), NFκB-regulated human IL22 receptor alpha unit (hIL22R), and stat3-driven mouse IL4 and IL10 (mIL4 and mIL10) genes. In psoriatic patients, high levels of TNF bind to its receptor and activate the expression of hIL22RA alpha via NF-κB. The newly synthesized hIL22RA is translocated to the membrane where it forms a functional heterodimer with IL10RB, initiating a signaling cascade that results in the phosphorylation of STAT3. The phosphorylated STAT3 forms a functional dimer that is translocated to the nucleus, resulting in enhanced expression of anti-psoriatic interleukins, mIL4 and mIL10.

Cytokines are peptide-based signaling molecules that play a major role in cellular communication and the immune response (Turner et al., 2014). Abnormal cytokine secretion contributes to many conditions, such as inflammatory and autoimmune diseases, cancer, and neurological disorders(Kany et al., 2019). For example, psoriasis is a chronic inflammatory skin disease characterized by the overproduction of pro-inflammatory cytokines, particularly tumor necrosis factor (TNF) and interleukin-2 (IL22) (Rendon and Schäkel, 2019). Because there is no cure, the main therapeutic approaches focus on symptomatic treatment, including the use of antibodies that neutralize pro-inflammatory cytokines and immunosuppressant drugs such as methotrexate and cyclosporine. Though these agents provide symptomatic relief, they are associated with severe side effects, such recurrent infections and immunogenicity. Recent clinical trials have demonstrated that the administration of immunomodulatory cytokines IL4 (22) and/or IL10 (23) at well-tolerated doses significantly improves psoriasis. Nevertheless, these interleukins are quickly eliminated from the blood circulation, necessitating frequent injections. Schukur et al. developed cytokine-converter designer cells for autonomous management of psoriasis (Schukur et al., 2015). In their system, an AND-logic gate was designed using TNFα and IL22 as signal inputs, and IL4 plus IL10 as a secreted output (Figure 5A). They engineered HEK-293T cells with TNFα receptor (TNFR) rewired to the expression of IL22RA subunit through the NF-κB pathway. The expressed IL22RA forms a heterodimer with endogenously expressed IL10RB subunit, which can be activated by IL22, employed as a second biomarker of psoriasis. The binding of IL22 initiates a signaling cascade through the JAK1/STAT3 pathway, leading to the expression and secretion of STAT3-driven mIL4 and mIL-10 into the extracellular space. These cells successfully treated psoriasis in a mouse model.

## Designer cells for cancer treatment

**Figure 6:**
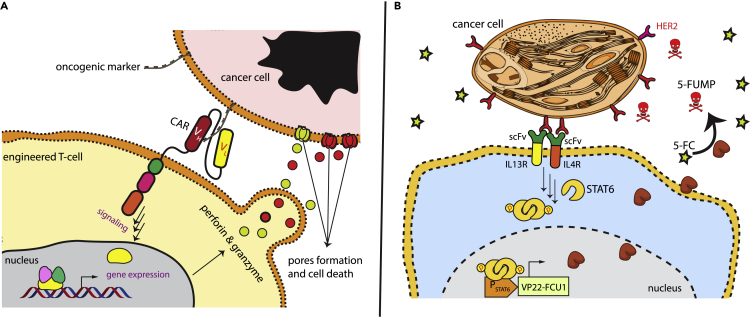
Designer cells for anticancer therapy (A) Schematic illustration of CAR-T cell technology. Autologous T-cells are equipped with a synthetic CAR receptor that recognizes an oncogenic cell-surface marker. After expansion, the engineered T cells are injected into the same patient. The designer cells bind to tumor cells through the CAR receptor and activate signaling pathways that trigger the secretion of cytotoxic proteins, such as perforins and granzymes, destroying the tumor cells. (B) The engineering of nonimmune cells for cancer eradication. HEK-293T cells were equipped with a synthetic receptor engineered by fusing the intracellular part of IL13R or IL4R to scFV against HER2 oncogene. VP22-FCU1 was also introduced under a STAT6 promoter. The binding of the synthetic receptor to HER2 molecules, which decorate cancer cell membranes, activates STAT6, which is translocated to the nucleus, resulting in expression of VP22-FCU1. VP22-FCU1 metabolizes the prodrug 5-fluorocytosine (5-FC) to the cytotoxic metabolite 5-FUMP, leading to local destruction of cancer cells.

The well-established chimeric antigen receptor (CAR)-T technology is a cell-based therapy that is clinically effective against certain types of malignancies, with long-lasting beneficial effects (June et al., 2018; Grupp et al., 2013). Autologous T-cells are isolated from patients and then genetically engineered to express synthetic CARs, which function to redirect T cells to selectively recognize and destroy cancerous cells bearing specified epitopes on their surface (Figure 6A). The FDA has approved the use of CAR-T cells to treat hematopoietic cancers. However, this system still has serious drawbacks, which limit its clinical use. These include life-threatening adverse effects and cell-associated toxicities, inadequate efficacy against solid tumors, the development of resistance, and antigen escape (Sterner and Sterner, 2021).

Nonimmune cells engineered with a synthetic T-cell receptor-like signal-transduction device have also been engineered and investigated as a potential anticancer therapy (Kojima et al., 2018). Kojima et al. designed therapeutic cells that recognize oncogenic markers on the cell membrane. They engineered a sensor-effector receptor consisting of a single-chain variable fragment (scFv) against HER2 oncogene fused to IL13R and IL4R heterodimer (Figure 6B). The binding of HER2 epitope to this heterodimer-based receptor induces a conformational change that results in activation of the JAK-STAT pathway. To reduce the leakiness of the system, the authors used a repressor protein – a CD43ex-45int chimera built from the extracellular and transmembrane domains of CD43 and the intracellular domain of CD45. The output gene in this gene circuit is VP22-FCU1, which encodes an enzyme that converts the anticancer prodrug 5-fluorocytosine (5-FC) into cytotoxic 5-FUMP, leading to local eradication of cancer cells.

## Conclusion and outlook

Designer cell-based therapeutic technology has already demonstrated its superiority over conventional drugs for the management of some difficult-to-treat diseases. An example of their clinical potential is provided by CAR-T cells, which are already in clinical use to cure various life-threatening hematopoietic tumors. Furthermore, autonomously operating designer cells would likely improve compliance and quality of life of patients with chronic diseases requiring invasive treatment, such as diabetes. However, a number of challenges remain.

The possibility of off-target induction (unexpected and undesired activation) of designer cells should be taken into account during their engineering for therapeutic purposes. The nature of the signaling circuit that connects the input-sensing transducer to effector modules has a significant impact on the performance and safety profile of designer cells. A totally orthogonal signaling cascade is generally required to build safe and reliable designer cells in order to rule out undesired induction. Orthogonal signaling refers to an ectopically introduced signaling network that is not influenced by endogenous pathways of the organism, thus ensuring tightly controlled and selective activation. Because many genetically encoded transducers are naturally wired to endogenous pathways, engineering of novel sensing transducers with the ability to orthogonally stimulate designer cells will be an important avenue for future research. Alternatively, engineering of designer cells with an AND-gate operation mode, that is, an activation mechanism which functions only in simultaneous presence of at least two different inducers, can be used to reduce off-target induction. However, the engineering of such logic gates is not an easy task, as it requires the availability of at least two different, reliable biomarkers for the same disease.

Open-loop operating expression systems can offer ON/OFF behavior, depending on the inducer availability in the cell environment. Although open-loop-based devices required repetitive administration of the inducer, a combination of open and closed loops may be very highly tunable, as well as potentially having the ability to temporarily block designer cell function if necessary or desirable. For example, if autonomous designer cells could be turned off by an external inducer, this would provide another control point in the event of therapeutic-induced toxicity or if the cell implant does not function as intended. Designer cells equipped with such an “emergency stop button” would offer a new layer of safety, especially for cell implants intended for long-term therapeutic use.

In fact, typical biomarkers are not available for all diseases, making the design of closed-loop gene circuits problematic. It has recently been demonstrated that specific extracellular RNAs (exRNAs) molecules are secreted into the body circulation in certain disease states (Xi et al., 2017). For example, several studies have characterized a number of distinctive exRNA molecules that are associated with Alzheimer’s disease, cancers, and numerous inflammatory diseases (Roser et al., 2018; Xu et al., 2021). Thus, the bioengineering of selective and sensitive cell-based molecular architectures that can detect and utilize these exRNAs as signal inputs would significantly increase the disease biomarker repertoire, facilitating the design of therapeutic designer cells for a wide range of diseases. Despite all the challenges that are still associated with their engineering, it is already clear that therapeutic designer cells will play a pivotal role in next-generation medicine.
